# Supernormal Conduction and Suppression of Spatially Discordant Alternans of Cardiac Action Potentials

**DOI:** 10.3389/fphys.2015.00407

**Published:** 2016-01-06

**Authors:** Linyuan Jing, Anuj Agarwal, Abhijit Patwardhan

**Affiliations:** Department of Biomedical Engineering, College of Engineering, University of KentuckyLexington, KY, USA

**Keywords:** supernormal conduction, conduction velocity restitution, discordant alternans, ventricular arrhythmia, action potential

## Abstract

Spatially discordant alternans (DA) of action potential durations (APD) is thought to be more pro-arrhythmic than concordant alternans. Super normal conduction (SNC) has been reported to suppress formation of DA. An increase in conduction velocity (CV) as activation rate increases, i.e., a negative CV restitution, is widely considered as hallmark of SNC. Our aim in this study is to show that it is not an increase in CV for faster rates that prevents formation of DA, rather, it is the ratio of the CV for the short relative to the long activation that is critical in DA suppression. To illustrate this subtlety, we simulated this phenomenon using two approaches; (1) by using the standard, i.e., S1S2 protocol to quantify restitution and disabling the slow inactivation gate j of the sodium current (I_Na_), and (2) by using the dynamic, i.e., S1S1 protocol for quantification of restitution and increasing I_Na_ at different cycle lengths (CL). Even though both approaches produced similar CV restitution curves, DA was suppressed only during the first approach, where the CV of the short of the long-short action potential (AP) pattern was selectively increased. These results show that negative CV restitution, which is considered characteristic of SNC, *per se*, is not causal in suppressing DA, rather, the critical factor is a change in the ratio of the velocities of the short and the long APs.

## Introduction

Ventricular arrhythmia is frequently preceded by alternans of action potential (AP) duration (APD), which is beat-to-beat alternation of APD. In certain circumstances, alternans initiated in one area can change from a long-short to short-long pattern in another area when traveling through the heart. This spatial change in pattern of alternans is called discordant alternans (DA). It has been shown that DA increases the dispersion of repolarization within the heart, and therefore, increases the probability of block and re-entrant circuit, both conditions being conducive to ventricular arrhythmia (Qu et al., 2000). Restitution of conduction velocity (CV) relates CV of an AP with either CL or its previous diastolic interval (DI) and is a critical mechanism in formation of DA (de Diego et al., 2008; Mironov et al., 2008). In most cases, the “normal” CV restitution exists where the slope of the restitution curve is either positive or zero, i.e., the CV decreases with increasing activation rate and for slower rates remains unchanged. The super normal conduction, SNC, which is paradoxically faster conduction of short beat and has been reported in several studies, is often represented by a negative CV restitution, which is a paradoxical increase in CV during fast activation rates (Chialvo et al., 1990; Davidenko et al., 1990). In most studies, SNC is quantified using the so-called standard S1S2 protocol under the conditions of reduced extracellular potassium concentration (Chialvo et al., 1990; Davidenko et al., 1990; Luo and Rudy, 1991; de Lange and Kucera, 2010). In the S1S2 protocol, the tissue is paced for N beats at constant cycle length (equal to S1) followed by a stimulus (S2) delivered at progressively shorter or longer intervals (i.e., the interval between the last S1 stimulus and S2 is varied and then the sequence of N stimuli at S1 interval is repeated). The CVs of the activations resulting from the S2 beat are graphed against the S1-S2 intervals to form the CV restitution curve. In simulations (de Lange and Kucera, 2010), SNC has also been produced at a normal potassium concentration by disabling the slow inactivation gate j of the sodium current (I_Na_). A recent study by Echebarria et al. (2011) showed that SNC can stabilize concordant alternans and prevent DA, although it has also been shown that SNC can amplify alternans amplitude and increase the possibility of block (de Lange and Kucera, 2009, 2010).

As stated above, SNC is generally characterized as an increase in CV at faster activation rates, i.e., a negative CV restitution. When SNC is observed during S1S2 protocol during the above discussed substrates, the S1S2 protocol implicitly focuses only on the shorter APD and shows a negative CV restitution because the velocity of the short AP increases relative to the long AP. However, this subtlety, i.e., the increase is predominantly for the short beat, is not always apparent. This depiction, therefore, leaves the potential for misinterpretation that the increase in CV at faster rates leads to suppression of DA. For example, during a substrate where both velocities increase in proportion at faster rates, the negative CV restitution could look exactly like that obtained during SNC, yet DA would not be eliminated. In the current study, we demonstrate the role of negative CV restitution in removing DA by producing a negative CV restitution in a simulated linear strand of cardiac tissue using two different approaches. Our results show that while similar negative CV restitution curves were observed in both situations, DA was only removed in the situation where CV of the short AP in a long-short pair was increased selectively. These results show that negative CV restitution, by itself, is not the reason why DA is prevented, rather it is a change in the ratio of the CVs of the short and long APs that is critical in removal of DA.

## Methods

The canine ventricular myocyte (CVM) model, developed by Fox et al. (2002) with the modifications reported by Hua and Gilmour (2004) was used for all simulations. Transmembrane potentials were simulated in a linear strand of 1000 cells. Pacing stimuli were applied to the fifth cell from one end. A no-flux boundary condition was used on both ends of the strand. Cell to cell coupling was modeled using diffusion. Diffusion coefficient was 7 × 10^−4^ cm^2^/ms and the simulated cell membrane capacitance was 1 μF/cm^2^. Customized code written in FORTRAN was used for implementation of the model.

Two pacing protocols were used to quantify CV restitution: standard and dynamic protocol. The dynamic pacing protocol, i.e., S1S1 protocol, is where the tissue is paced for N beats at constant cycle length (S1), and then this process is repeated by progressively shortening S1-S1 intervals. The APD and the preceding DI from the last S1 stimuli at any CL are used to compute restitution.

1. Standard S1S2 protocol, where the S1-S1 interval was 250 ms and the S1-S2 interval was progressively decreased from 500 to 200 ms with a decrement of 50 ms and from 200 ms, the decrement was 5 ms until block occurred.
2. Dynamic S1S1 protocol (Koller et al., 1998), where pacing started with a CL of 500 ms and once steady state was obtained, the CL was decreased in the same decrements as the S1-S2 interval in standard protocol. The strand was paced for 90 beats at each CL until block occurred. CV at each CL was calculated as the average velocity of the last two APs at that CL, i.e., average of the long and the short AP.

Two approaches of I_Na_ modification were used to create negative CV restitution: (1) the slow inactivation gate j of I_Na_ was disabled, i.e., it was set to a constant value of 1 for all cells, which is the same method that was used by de Lange and Kucera (2010); (2) I_Na_ was manipulated (increased) at each CL by a different amount.

Three different combinations of the protocols and Ina modifications were used to compare their effects on DA:

Protocol 1: standard protocol + disabled j gate (*j* = 1);
Protocol 2: dynamic protocol + increasing I_Na_ at each CL;
Protocol 3: dynamic protocol + disabled j gate (*j* = 1).

To facilitate a direct comparison between the two approaches, for Protocol 2 we adjusted the change in I_Na_ at each CL such that the resulting negative CV restitution was very similar to the one generated using Protocol 1.

In order to determine the effect on DA, at each CL, the strand was paced for 90 beats to obtain steady state. If DA was observed at a particular CL, then the simulation was run for an additional 90 beats with the I_Na_ modification that was used to obtain negative CV restitution, i.e., either by disabling the j gate or by increasing I_Na_. APDs were computed at different sites (cells) to determine existence of DA and whether it diminished, or disappeared after the modification. Time-space plots were used to visualize the effects of the modifications on conduction.

All analysis was conducted in MATLAB (Mathworks, Natick MA) using custom developed programs. APDs were computed as the duration between the start of an AP and its 90% repolarization. CV was determined as the ratio of distance (in centimeters) between cell 25 and 75 and the time delay between the onsets of AP (in seconds) at these cells. In case of the standard protocol, by definition, restitution was quantified using the CV of the AP resulting from the S2 stimulus. In case of the dynamic protocol, the restitution was quantified using the average of the CV of last two APs at each CL.

## Results

**Figure 1:**
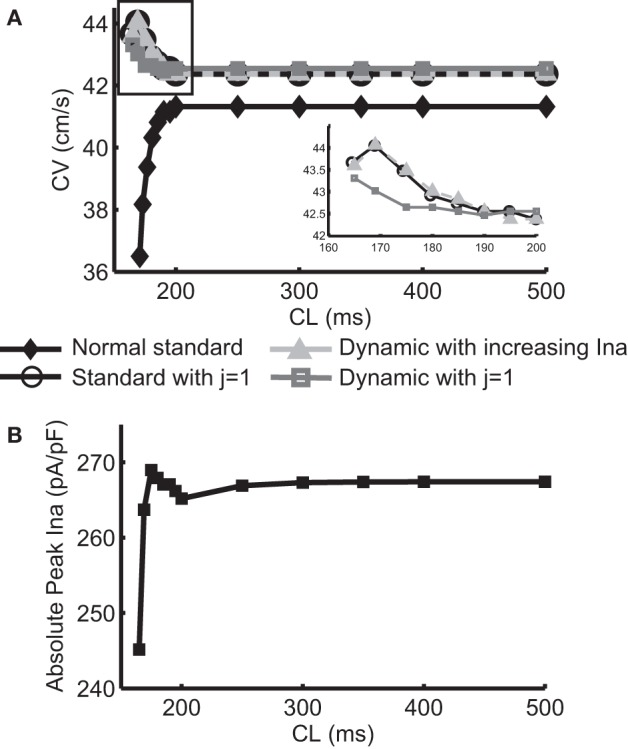
**(A)** Normal CV restitution obtained using the standard S1S2 protocol overlaid with negative CV restitutions obtained using three different approaches. The inset shows a “zoomed in” view of the three negative CV restitutions at CL ≤ 200 ms. **(B)** Trace of peak I_Na_ (absolute values) at each CL used in protocol 2.

Figure 1A shows the normal CV restitution obtained using a normal (i.e., no I_Na_ modification) standard protocol, and the negative CV restitutions obtained using the three protocols described in the methods section. The normal CV restitution was as expected: as CL decreased from 500 to 200 ms, change in CV was minimal, however, when CL was below 200 ms, CV decreased rapidly. Activation block occurred at CL < 165 ms. The inset in Figure 1A shows an overlay of the three negative CV restitution curves constructed by using the three different protocols when CL ≤ 200 ms. As shown in the figure, instead of a relationship with steep positive slope as was the case during normal conduction, in these three cases, the slopes of the restitution curves became negative for CL < 200 ms, and for Protocol 1 and 2, the CV restitution became positive again for CL < 169 ms. This behavior of the CV restitution, i.e., increasing CV with increasing activation rate, is considered to be characteristic of SNC. In Protocol 2, we selected the level of change in I_Na_ at each CL to match the values of CV to those resulting from Protocol 1. The peak I_Na_ at each CL (absolute values) is shown in Figure 1B. A close match between the two negative CV restitution curves produced by protocol 1 and 2 is seen in the figure.

Table 1 includes a summary of the APD values and alternans amplitudes at the pacing site (cell 5) during normal conduction and during the two I_Na_ modifications for CL ≤ 190 ms. In default conditions, i.e., neither j gate was disabled nor I_Na_ was elevated, APD alternans initiated at CL = 190 ms. Compared to amplitude of APD alternans during normal conduction, the alternans amplitude near the proximal end remained more or less the same when j gate was set equal to 1 (Protocol 1 and 3), and it was smaller when I_Na_ was increased (Protocol 2). For each CL, APDs at different cell locations along the strand were computed to determine the existence of DA. During normal conduction, DA occurred when CL ≤ 185 ms. Disabling the j gate (Protocol 1 and 3) amplified APD alternans amplitude at the distal end, but suppressed the DA within 50 beats at all CLs ≤ 185 ms. Increasing I_Na_ (Protocol 2) decreased the APD alternans amplitude at all sites, but the DA existed, also for all CLs ≤ 185 ms.

**Table 1:** **Summary of APDs (in milliseconds) during normal conduction (NC) and SNCs at different CL**.

**CL (ms)**	**NC**			**Increasing I**_**Na**_			**Disabled j gate**		
	**APD odd**	**APD even**	**ΔAPD**	**APD odd**	**APD even**	**ΔAPD**	**APD odd**	**APD even**	**ΔAPD**
190	146.9	142.1	4.8	147.5	146.1	1.4	143.7	143.7	0
185	152.8	126.1	26.7	155.1	128	27.1	148.8	131.6	17.2
180	154.7	113.7	41	156.8	115.8	41	151.4	118.5	32.9
175	155.6	103	52.6	157.5	105.8	51.7	151.7	109.1	42.6
169	155.8	91.5	64.3	157.9	95	62.9	151.4	99.7	51.7
165	Block			158.1	88.5	69.6	152	92.6	59.4

**Figure 2:**
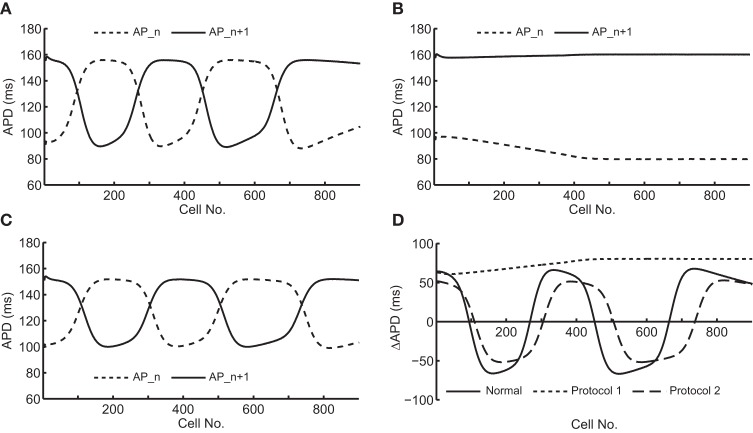
**Spatial changes in APDs of an alternating pair at CL of 169 ms during the following situations: (A) normal conduction; (B) j gate was set equal to 1; (C) I_Na_ was increased by 23%. (D)** shows an overlay of the amplitudes of APD alternans during the three situations.

**Figure 3:**
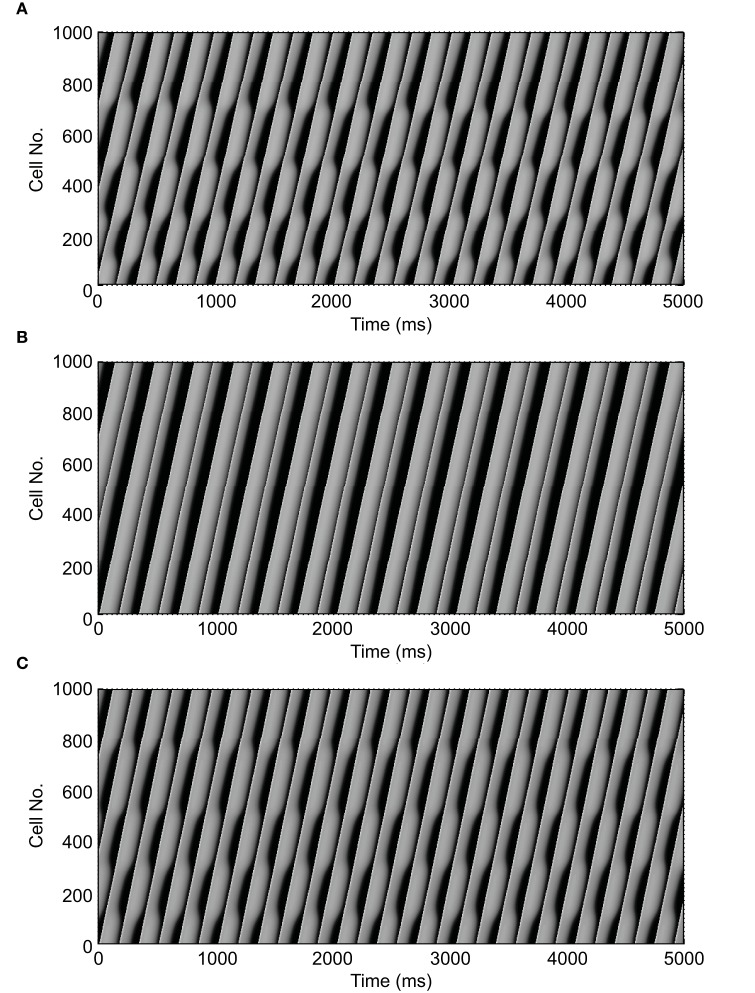
**Time-space plots of the last 30 beats at CL of 169 ms during normal conduction and the two situations of SNC**. The figure shows that discordant alternans formed during normal conduction **(A)** and that it was eliminated when gate j was set equal to 1 **(B)**, but not when I_Na_ was increased for all beats **(C)**. Note that both circumstances, in **(B,C)**, had negative CV restitution. **(A)** is reproduced from our previous study (Jing et al., 2012).

Figure 2 shows an example of spatial changes in APD for a propagating AP at CL of 169 ms. Shown are the last two APs during normal conduction (Figure 2A), disabled j gate (Figure 2B), increased I_Na_ (Figure 2C) and the alternans amplitude during these situations (Figure 2D). The effects of the two I_Na_ modifications are shown in Figures 3B,C as time-space plots. Both panels (Figures 3B,C) show the last 30 of the total 90 beats at CL of 169 ms. Under normal conduction, i.e., the default condition of the model, concordant alternans first transitioned into DA at around cell 100 (Figure 3A) and several nodes were formed in the strand. As stated above, when the j gate was set to 1, DA went away within about 50 beats and concordant alternans persisted through the rest of the simulation, as shown in Figure 3B. However, when I_Na_ was increased for all beats the DA persisted (Figure 3C) despite a negative slope of CV restitution (Figure 1A, gray dashed line with filled triangles).

**Figure 4:**
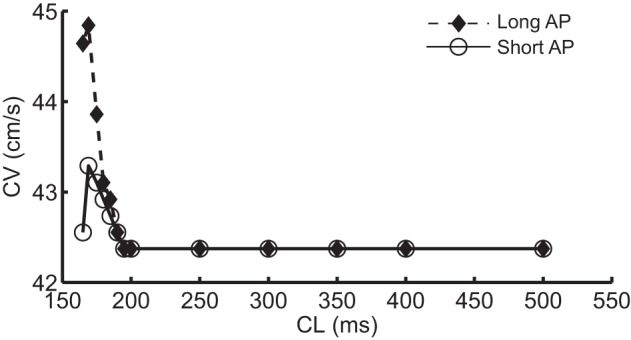
**Restitution curves during alternans computed separately using long AP (dashed line with filled diamonds) and short APs (solid line with open circles) during the dynamic protocol**.

To determine the difference between the CVs of the long and short APs during alternans, for the dynamic protocol with increased I_Na_ (Protocol 2), we plotted the restitution curves using CVs of odd (long) and even (short) APs separately, these curves are shown in Figure 4. The figure shows that during this modification, when alternans occurred, CVs of both short and long APs were higher at shorter CL, however, the amount of increase was more for the long APs. Table 2 summarizes the ratios of CVs of the long and short APs at each CL during the two negative CV restitution conditions. Consistent with the restitution curves shown in Figure 4, during Protocol 2, the ratios were ≤ 1, i.e., the CV of the long AP was always larger than the CV of the short AP. However, when *j* = 1 (Protocol 1 and 3), the ratios were ≥ 1, showing that the short AP traveled faster compared to the long AP.

**Table 2:** **Ratio of CVs for short AP and long AP during alternans (CL ≤ 190 ms)**.

**CL (ms)**	**Ratio: CV_S_/CV_L_**	
	**Increasing I_Na_**	**Disabled j**
190	1	0.996
185	0.996	1
180	0.996	1.004
175	0.983	1.013
169	0.965	1.031
165	0.953	1.044

*The subscripts for CV denote the velocity of the short or long AP*.

**Figure 5:**
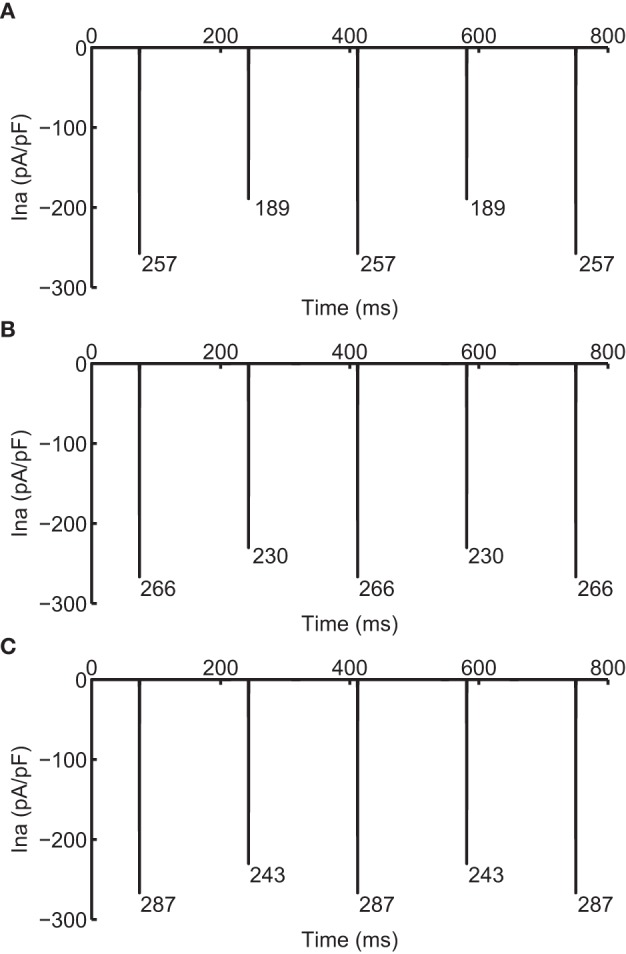
**I_Na_ during normal (A) and supernormal (B,C) conduction**. **(B)** shows I_Na_ when j gate was set equal to 1 and **(C)** shows that when the computed I_Na_ was increased for all beats by 23%, i.e., computed I_*Na*_ was scaled by 1.23. All currents are shown at CL of 169 ms. The number below each negative peak indicates the peak I_Na_ value for that AP.

As CV is affected by the availability of I_Na_, we recorded the change in I_Na_ under the two different modifications that resulted in negative CV restitution. Figure 5 shows I_Na_ during normal conduction and during negative CV restitution at CL of 169 ms. During normal conduction (Figure 5A), I_Na_ alternated beat to beat following the same long-short pattern of APD alternans, i.e., I_Na_ was larger for the long AP than that for the short AP. While during the two circumstances of negative CV restitution (Figures 5B,C), although the alternating pattern was also present, I_Na_ showed either a predominant increase at the onset of the short AP (Figure 5B), or a substantial increase at the onset of both long and short APs (Figure 5C). In both cases (Figures 5B,C) there was an overall increase in the availability of I_Na_ compared with that during normal conduction (Figure 5A). As a result, the CV at this CL was higher than the CV at a longer CL, consistent with the criteria for negative CV restitution. We also computed the ratio of I_Na_ between the short and long AP at each CL (Table 3) during the two situations. Although in both cases the ratio remained ≤ 1 (I_Na_ for short AP is less than I_Na_ for long AP), the ratio increased when j gate was set to 1, suggesting a predominant increase in I_Na_ for the short AP, and therefore, more increase in CV for short AP.

**Table 3:** **Ratio of I_Na_ for short AP and long AP during alternans (CL ≤ 190 ms)**.

**CL (ms)**	**Increasing I**_**Na**_			**Disabling j gate**		
	**I_Na_ short**	**I_Na_ long**	**Ratio**	**I_Na_ short**	**I_Na_ long**	**Ratio**
190	267	267	1.00	265	266	1.00
185	264	270	0.98	263	266	0.99
180	261	274	0.95	261	267	0.98
175	257	281	0.92	254	267	0.95
169	240	288	0.83	229	267	0.86
165	204	287	0.71	217	267	0.81

## Discussion

**Figure 6:**
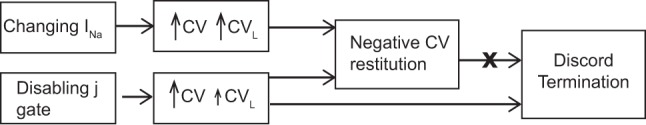
**Schematic showing the mechanistic link between SNC, generated using different methods, and discordant alternans**. Note that the size of the arrows indicates the magnitude of the change in CV. For the case of disabled j gate, the increase in the CV of the short AP was more than that for the long AP.

The main finding of our study is a demonstration that the mechanism by which DA is eliminated during SNC is actually selective preservation of the CV of the short AP and not negative CV restitution *per se*. As reported previously by others (Echebarria et al., 2011), our results also show that when SNC was present, DA did not occur. The key issue, however, is that DA was eliminated only when the negative CV restitution was obtained using very specific circumstances and not in general. The previous studies report that SNC as indicated by a negative CV restitution eliminated DA, the caveat, that it is so only when negative CV restitution is observed during SNC is not always apparent and this leaves room for misinterpretation that negative CV restitution is responsible for suppression of DA. Our results show that instead of what is considered the characteristic of SNC, i.e., the negative CV restitution, the mechanism behind suppression of DA is a relative change in CV of the long and the short beats, regardless of the pacing protocol (standard or dynamic, as shown in Protocol 1 and 3) used to characterize the restitution. Further, not all circumstances causing negative CV restitution may satisfy the condition required to suppress DA (as shown in Protocol 2). A schematic, in Figure 6, summarizes the above scenario.

SNC has been observed in several studies, in most cases, it is quantified using a standard S1S2 protocol which only includes velocity of short beat (Chialvo et al., 1990; Davidenko et al., 1990; Luo and Rudy, 1991; de Lange and Kucera, 2010). A recent study by Echebarria et al. (2011) reported that concordant alternans were stabilized during SNC, and DA only occurred during normal conduction but not during SNC. Part of our results are similar to their observations to the extent that we also observed DA during normal conduction and that it was not present when negative CV restitution resulted by disabling the slow inactivation gate j of I_Na_ (Figure 3B). This method is the same method as that used by de Lange and Kucera (2010) to simulate SNC, and our results of amplification of alternans amplitude at the distal end of the strand (Figure 2D) under these conditions were also consistent with de Lange et al's observations.

As discussed previously by others (Qu et al., 2000; Weiss et al., 2000; de Diego et al., 2008; Mironov et al., 2008; de Lange and Kucera, 2010), concordant alternans transition to DA when higher CV of a long AP leads to shortening of its preceding DI as the impulse travels distally, which further leads to shortening of the APD and decrease in CV. Eventually, the long AP becomes a short AP, thus setting up DA. The opposite happens for the conduction of a short AP. During SNC, as the CV of the short AP is increased, the lengthening of the preceding DI and the short AP is prevented. Instead, the APD of a long AP becomes even longer and the APD of a short AP becomes shorter. As a result, the concordant alternans is preserved. The above mechanism explains how SNC produced by disabling the j gate (Protocol 1 and 3) prevents DA.

The ionic mechanism of SNC preventing DA lies in the selective change of I_Na_: CV is critically affected by the maximum rate of depolarization, which is determined by the availability of I_Na_. Disabling gate j (Protocol 1 and 3) essentially increases the I_Na_ that is available before the start of an AP, and therefore, increased the CV of that AP. In a standard S1S2 protocol, the effect is predominantly on the S2 beat, because the longer DI before the S1 beat allows almost all I_Na_ channels to recover from the previous activation. Conversely, the DI preceding the S2 beat is short so less I_Na_ is available. The shorter the CL, the less the I_Na_ available for S2 beats. Thus, by setting the j gate equal to 1, CV of the S2 beat is increased more compared to the S1 beat and it is also increased more compared to the S2 beat with a longer CL (i.e., the previous S1-S2 pair). The same explanation holds during alternans at any CL. The ratios in Table 3 show the predominant I_Na_ increase for the short AP (also shown in Figure 5 at CL = 169 ms). The selective effect of change in I_Na_ resulted in the CV of the short AP to exceed that of the long AP, as shown in Table 2. Further confirmation of the effect of selective increase in I_Na_ is provided by results of simulations that we reported in a previous study (Jing et al., 2012). Briefly, in that simulation once DA was established in the strand, APD for a beat to occur was predicted “on the fly” and when it was predicted to be short, the I_Na_ for that AP was increased by a factor of 1.8. This approach allowed selectively increasing the I_Na_ for short APs only. Results of this simulation, in Figure 5 of that study, showed that selective increase in I_Na_ for the short AP eliminated DA. In that study the ratios of the CVs (long/short) were 41.3/39.2 cm/s and changed to 41.3/46.9 cm/s during selective increase of I_Na_ which also eliminated DA.

On the other hand, when we used a dynamic protocol with increase of I_Na_ (Protocol 2) to generate a negative CV restitution, this condition did not eliminate DA. In this situation, I_Na_ was increased for both long and short APs by the same amount, so the ratio of I_Na_ between short and long AP was preserved (Table 3, Figure 5). Therefore, CV for both long and short APs increased proportionally, and CV of the long AP remained higher than CV for the short AP. As a result, the DA was not eliminated.

The physiological conditions when the phenomenon similar to “disabling the j gate” would occur is not known at this point. However, during *in vitro* experiments using tissues from pigs and dogs, we have shown that the maximal rates of depolarization for a short AP can be larger than that for the long AP (Jing et al., 2012), which suggests that selective increase in the CV of the short AP, relative to the long AP is possible and may occur spontaneously. The exact mechanism via which this occurs is unclear and needs further investigation.

It has been shown that SNC could be produced by manipulating extracellular potassium, however, we were not able to reproduce the phenomenon in our model. This lack may be due to differences in the parameters used by us and the other group, however these differences also remain unclear at this point.

We acknowledge that the selective increase in I_Na_ that we used to produce negative CV restitution (Protocol 2) is a synthetic approach and was primarily employed to demonstrate the point that increasing CVs for both long and short beats proportionately at faster activation rates does not suppress DA. This purely synthetic approach was adequate for our purposes, because our goal was not to focus on what causes SNC, rather it was on what SNC causes in terms of its effects on DA. However, there is some experimental evidence that such scenario may be possible in myocytes. A previous study (Wang et al., 2000) of the voltage dependent potassium channel Kv1.5 has shown supernormal Na^+^ conductance during recovery from one of the inactivation states (R state) in the repolarization phase of an AP. That is, during the R state, this potassium channel becomes more conductive to Na^+^ than to K^+^, suggesting how increased I_Na_ could occur at short CL. The Kv1.5 is primarily an atrial channel. Whether similar phenomenon exists in the ventricles is unknown, however these observations suggest that such scenario is not physiologically impossible.

## Conclusion

In summary, although the results in the literature suggest and imply that the characteristic of SNC, i.e., negative CV restitution, is a mechanism that suppresses discordant alternans, our results show that the key factor in eliminating discordant alternans is not the negative CV restitution *per se*; instead, it is the preservation of the velocity of the short AP in an alternating pair that plays the critical role.

## Funding

Supported in part by grants from the National Science Foundation (0730450, 0814194) and the Commonwealth of Kentucky.

### Conflict of interest statement

The authors declare that the research was conducted in the absence of any commercial or financial relationships that could be construed as a potential conflict of interest.
